# Editorial: Women's career motivation

**DOI:** 10.3389/fpsyg.2024.1390333

**Published:** 2024-06-04

**Authors:** Norita Ahmad, Linzi Kemp, Jana Fedtke

**Affiliations:** ^1^Department of Marketing and Information Systems, School of Business Administration, American University of Sharjah, Sharjah, United Arab Emirates; ^2^Department of Management, School of Business, American University of Ras Al Khaimah, Ras Al Khaimah, United Arab Emirates; ^3^Department of Liberal Arts, Liberal Arts Program, Northwestern University in Qatar, Doha, Qatar

**Keywords:** women, motivation, career, leadership, workplace

In this Research Topic, we highlight the importance of women's career choices in contemporary societies. Empowering women in diverse professional fields contributes to diversity, innovation, and economic growth. It also fosters equality as it challenges gender stereotypes, which leads to societal progress. The articles in this Research Topic probe into multifaceted challenges confronted by women in the workplace and offer potential strategies for navigating and overcoming these barriers.

The papers featured in this Research Topic offer a comprehensive examination of the diverse experiences and challenges faced by women in their professional journeys:

Koekemoer et al. “*The subjective career success of women: the role of personal resources*” examines the role of resilience and grit in the relationship between women's person-environment fit and perceptions of career success in South Africa. Grounded in the Job Demands Resources Model and social cognitive theory, a cross-sectional online survey involving 408 female employees finds evidence that indicates significantly positive associations between person-environment fit, resilience, grit, and subjective career success. While resilience and grit mediate this relationship, their moderating effects could not be established. The findings contribute to understanding how personal resources impact women's career success, highlighting resilience and grit as vital mechanisms in overcoming workplace challenges.

Pillay-Naidoo and Vermeulen examine the experience of female leaders seeking solidarity in male-dominated sections within South Africa in their article titled, “*Seeking support through solidarity: female leader's experiences of workplace solidarity in male-dominated professions*.” It addresses the dearth of literature on strategies and motivating resources employed by female leaders to navigate workplace challenges. Despite extensive documentation of barriers, the study focuses on motivating factors aiding women in achieving leadership positions. Female solidarity emerges as a crucial motivating resource, countering the “queen bee syndrome.” Employing a qualitative approach with 13 semi-structured interviews of female leaders in South Africa's male-dominated professions, thematic content analysis identifies barriers (unfair behaviors, generational beliefs, societal expectations, and stereotypes), benefits (mentorship, recognition, female solidarity and support), and interventions (networking, cultural transformation, mentorship) related to female solidarity.

In “*Women's career motivation: social barriers and enablers in Sudan*,” Mohamed et al. highlight women's career choices in the North African context, specifically in Sudan, an often-neglected country in the Middle East and North Africa region (MENA). The study identifies challenges that hinder Sudanese women's career progression and limit their opportunities for growth. Employing a multifaceted approach, the study integrates psychological and organizational theories, benchmarking, and empirical investigations through a Delphi study involving 75 Sudanese women leaders. Results reveal the complex factors that women in Sudan face when making their career choices, emphasizing the unique impact of Sudan's social context on women's career motivations. The research offers actionable policy recommendations for Sudan and serves as a model for gender-responsive policies in similar MENA countries and beyond.

Finally, Chan and Hutchings draw attention to women with disabilities, an understudied minority, in their article on “*Inequalities, barriers, intersectionality, and facilitators of careers of women with disabilities: Themes and future research agenda from a scoping review*.” This contribution investigates the career motivations, opportunities, and development of women with disabilities. Existing research reveals a disability pay gap and lower representation in managerial and professional roles, often attributed to prejudice, discrimination, and stigmatization. Globally, there is an estimated one billion people with disabilities, but women face additional disadvantages due to their gender. The literature review identifies key themes such as career inequalities, barriers, educational interventions to improve career motivations and opportunities for women with disabilities, facilitators for career development of women with disabilities, and intersectionality of gender, disability, and other identities for women's career development.

Despite notable progress in reducing inequalities over the years, women continue to face significant disparities in their professional lives. In this context, understanding women's career motivations and fostering their career ambitions emerge as fundamental to promoting personal achievement and creating more inclusive and vibrant communities. The assortment of articles in this issue provides a glimpse into the diverse viewpoints on women's career motivations. There is ample scope for further research to delve into different geographic areas or to consider the experiences of additional minority groups, thereby broadening our understanding and support for women's career aspirations across various contexts.

## Author contributions

NA: Writing – original draft, Writing – review & editing. LK: Writing – original draft, Writing – review & editing. JF: Writing – original draft, Writing – review & editing.

